# T114. SCHIZOTYPAL PERSONALITY QUESTIONNAIRE-BRIEF: FACTOR STRUCTURE ANALYSIS IN A NONCLINICAL ROMANIAN SAMPLE

**DOI:** 10.1093/schbul/sby016.390

**Published:** 2018-04-01

**Authors:** Aziz Ferchiou, Maria Ladea, Mihai Bran, Marion Leboyer, Ruxandra Slavu, Franck Schurhoff, Andrei Szoke

**Affiliations:** 1 INSERM; 2 University of Medicine and Pharmacy “Carol Davila”; 3 H Mondor Hospital, DHU Paris-Est University, Fondation FondaMental; 4 Clinical Hospital of Psychiatry; 5 H Mondor Hospital, Fondation FondaMental

## Abstract

**Background:**

The study of schizotypal traits in the general population has been proposed as a way to understand aetiology and pathophysiology of schizophrenia. Self-report measures of psychometric schizotypy like the Brief version of the Schizotypal Personality Questionnaire (SPQ-B) have been shown to be valid, inexpensive and non-invasive tools. Few studies used a Likert-type scale format, which could be better able to allow partial endorsement and to detect more defended respondents than the forced choice format. At our knowledge, no studies of the SPQ-B used validity and social desirability items, to assess the potential impact of random or biased answers.

**Methods:**

We examined factor structure and internal reliability of a Romanian version of the Schizotypal Personality Questionnaire-Brief (SPQ-B), in a Likert format in a sample of 580 students of Universities of Bucarest, Craiova and Brasov, in Romania. 3 validity items and 5 items of social desirability were added to the 22-items SPQ-B. We investigated the dimensional structure of the Romanian version of the SPQ-B first in the entire sample, and then after eliminating “bad” responders (i.e. those with aberrant answers on the validity items). We used a Principal Components Analysis (PCA) followed by a promax rotation. Factor selection was based on Eigenvalues over 1.0 (Kaiser’s criterion), Cattell’s scree plot test, and interpretability of the factors. We calculated Cronbach’s Alpha for total SPQ-B and for each dimension.

**Results:**

Our sample was constituted of 433 women and 147 men. The mean age was 25.5 ± 4.5 years. SPQ-B Likert total scores ranged from 23 to 90 points (mean = 55 ± 12). 71 participants were excluded after taking account of validity questions. Factor analysis of the entire sample resulted in a 3-factor solution that explained 43.8% of the variance. Factor 1 (Cognitive-perceptual; 10 items) includes items related to “ideas of reference”, “magical thinking”, “unusual perceptual experiences” and “suspiciousness”. Factor 2 (Interpersonal; 5 items) includes items related to “social anxiety”, “no close friends”, and “constricted affect”. Factor 3 (Disorganized; 7 items) includes items related to “odd behavior” and “odd speech”.

Coefficient Alpha for the three subscales and total scale, respectively, were 0.74, 0.78, 0.78 and 0.86. There were no significant differences when the analyses were conducted in the sample of 509 “good” responders’ students.

**Discussion:**

Factor analysis of the Romanian version of the SPQ-B in a Likert format confirmed the three-factor structure of schizotypy. The SPQ-B and its subscales demonstrated good internal reliability. The use of items of validity and social desirability did not change significantly the results.

